# A safe and successful capsulorhexis technique for the intumescent cataracts; modified two-stage continuous curvilinear capsulorhexis

**DOI:** 10.1186/s12886-023-02895-4

**Published:** 2023-04-04

**Authors:** Raşit Kılıç, Şerife Gülhan Konuk, Alper Güneş, Sebile Üstün Çomçalı

**Affiliations:** 1 Department of Ophthalmology, Faculty of Medicine, Tokat Gaziosmanpaşa University, Kaleardı Mah, Muhittin Fisunoğlu Cad. Tıp Fakultesi, Tokat, 60250 Turkey; 2 Department of Ophthalmology, Ankara Province Hospital, Ankara, Turkey

**Keywords:** Capsulorhexis, Cataract, Intumescent cataract, Phacoemulsification

## Abstract

**Background:**

Capsulorhexis is the most important step in intumescent cataract due to the high risk of radial extension of the capsular tear during the cataract surgery. The aim of this study is to present modified the two-stage capsulorhexis technique for intumescent cataract.

**Materials and methods:**

The two-stage capsulorhexis technique was used in this study. A small size capsulorhexis approximately 1.5-2 mm diameter was created in the first stage. Liquefied cortex was aspirated with a 25 G cannula to equalize anterior chamber pressure and intracapsular pressure after the small size capsulorhexis. In the second stage, a 5–6 mm capsulorhexis size was performed for a safe phacoemulsification.

**Results:**

A total of 73 consecutive patients with intumescent cataract were evaluated in this study. There were 39 male cases and 34 female cases. Mean age was 66 years ± 8 (between 53 and 84 years). A well centered complete continuous curvilinear capsulorhexis approximately 5–6 mm size was achieved in 72 of 73 cases (98.6%). Peripheral extension of capsulorhexis occurred in one eye during the second stage capsulorhexis. In this case, the capsule was cut with Vannas scissors and the capsulorhexis was completed. The rest of surgery was continued with a standard procedure and in-the-bag IOL implantation was done.

**Conclusions:**

This technique facilitates the creation of a safe capsulorhexis compared to the one-stage capsulorhexis technique. Surgeons may consider this technique to perform a safe phacoemulsification in the intumescent cataracts.

**Supplementary Information:**

The online version contains supplementary material available at 10.1186/s12886-023-02895-4.

## Introduction

Patient with intumescent cataract is one of the most challenging cases for the surgeons regardless of their experiences. Liquefaction of the cortex causes increased intracapsular pressure in these cases. Capsulorhexis is the most important step in this type of cataract due to the high risk of radial extension of the capsular tear.

The key point in intumescent cataracts is that the sudden outflow of intracapsular liquefied cortex due to high intralenticular pressure causes radial tear. For this reason, high and stable anterior chamber pressure should be obtained, which can resist the intralenticular pressure before the puncture of the capsule. Reducing the anterior capsule tension is very important for controlling the direction of the capsulorhexis flap. This can be achieved with an anterior chamber maintainer, infusion cannula, or cohesive ophthalmic viscoelastic device (OVD) [1–3]. Once this is achieved, a small capsular tear or capsulorhexis can be performed to reduce complications. Otherwise, the Argentinian flag may develop rapidly by puncturing the capsule before even the first aspiration can be made. Therefore, it is very critical to be able to create a tamponade effect that resists high intralenticular pressure. Even with a small initiating nick, the Argentinian flag sign may occur in cases of high intumescence. Those cataracts are the most challenging cases to deal with. For these cases, immediate flap creation and rotation with change in vector forces is essential to complete capsulorhexis. Other techniques such as phaco capsulotomy, cannula-vacuum technique can be successful after a high and stable anterior chamber pressure [4–10].For the visualization of the anterior capsule, trypan blue is generally used for staining but this increases the stiffness of the anterior capsule. This may also facilitate radial extension of the capsular tear [4, 5]. If the tear extends to the periphery, serious complication rates may increase such as posterior capsular tear, nucleus drop, vitreous loss and posterior dislocation of the intraocular lens.

There are studies in the literature including different techniques to prevent the extension of capsular tear towards periphery and to perform a safe cataract surgery in cases with intumescent cataract [4–10]. One of them is two-stage continuous curvilinear capsulorhexis [6, 11–13]. A small capsulorhexis size is performed in the first stage and liquefied cortex is aspirated in this technique. In the second stage, a normal size capsulorhexis is created. In this study, we modified the two-stage capsulorhexis technique for a safe surgery in the intumescent cataracts.

## Materials and methods

This retrospective study was conducted between June 2020 and July 2022 at the Department of Ophthalmology, Faculty of Medicine, Tokat Gaziosmanpasa University. The Helsinki Declaration Principles were followed and approval was obtained from the Ethics Committee of Gaziosmanpasa University. A voluntary informed consent was obtained from all patients. The files of patients who were operated with the modified two-stage continious curvilinear capsulorhexis technique due to intumescent cataract were evaluated retrospectively.

Patients with intumescent cataract were included in the study. Patients with subluxated lens, other types of cataract, a history of trauma and posterior segment pathologies such as retinal detachment were excluded from the study. All cases with intumescent cataract underwent a complete ophthalmic examination. A detailed history was queried from all participants. The examinations consisted of best corrected visual acuity (BCVA) evaluation with the Snellen chart, intraocular pressure measurement with an air puff tonometer, anterior segment examination with a slit lamp biomicroscopy, posterior segment evaluation with an ocular B-mode ultrasonography. Intumescent cataract diagnosis was based on shallow anterior chamber compared to fellow eye and lens swelling on slit‑lamp examination and confirmed by an A-scan US (US-4000, NIDEK CO. LTD., JAPAN). Intumescent cataracts show the presence of multiple internal acoustic reflections on A-scan US. Anterior chamber depth and lens thickness measurements were performed by A-scan US.

### Surgical technique

All surgeries were performed under topical anesthesia by the same surgeon (RK). Mannitol or any hyperosmolar agent was not used for the cases before all surgeries.

**Fig. 1:**
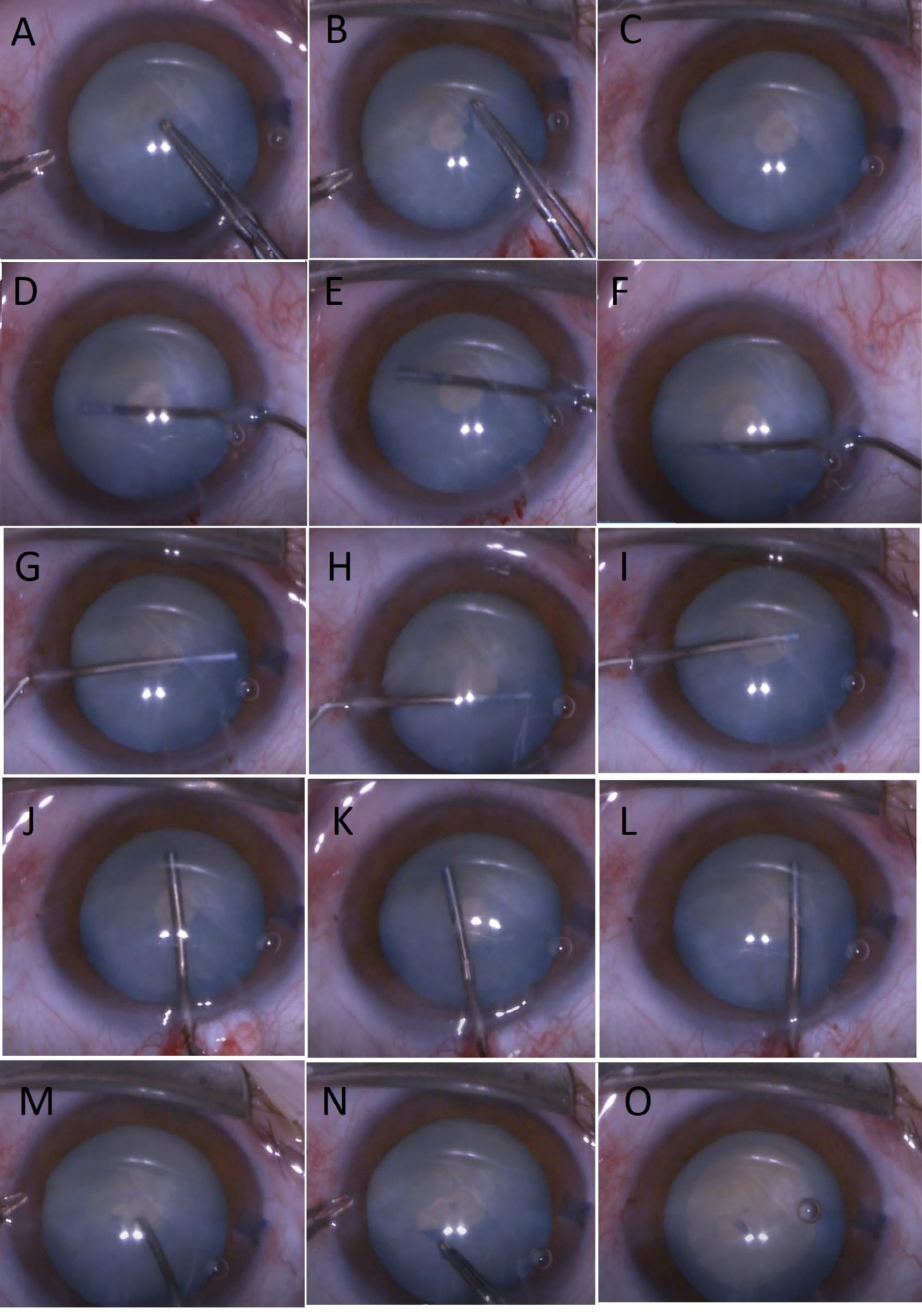
A-C: The first stage capsulorhexis is performed. An utrata is used during both capsulotomy and the first stage capsulorhexis D-L: Intracapsular liquefied cortex is aspirated with a 25 cannula. M-O: Capsulotomy is performed with a cystotome and the second stage capsulorhexis is created with an utrata Video 1 : The first stage capsulorhexis is performed as small as possible. The first stage capsulorhexis, aspiration of liquefied cortex with a 25 G cannula and creation of the second stage capsulorhexis are well seen in the video

All patients underwent small-incision cataract surgery with phacoemulsification technique. The single-plane side-port incisions were placed at 9 o’clock and 3 o’clock with a 21 G steel keratome. Trypan blue was injected to the anterior chamber to stain the anterior capsule under air. The anterior chamber was filled with cohesive OVD (CrownVisc 3.0% Na-hyaluronate, Miray Medical, Bursa, Turkey) using a side-port. The one-step clear corneal main incision was carried out at 12 o’clock using a 2.75 mm wide steel keratome. A cystotome was not used for puncturing the anterior capsule. Utrata was used for puncturing the anterior capsule and creating the first stage capsulorhexis as small as possible, approximately 1.5-2 mm diameter. Utrata was not withdrawn from the anterior chamber in both puncturing the anterior capsule and creating the first stage capsulorhexis to prevent leakage from anterior chamber and to maintain a stable anterior chamber pressure. If the blurring of the anterior chamber interfered with performing the first stage capsulorhexis, OVD was used to clear the anterior chamber and then capsulorhexis was completed. All liquefied cortex was aspirated manually using a 25 G cannula connected to a 5-mL syringe (Fig. 1). Additional OVD was injected to the anterior chamber through the main incision after aspirating liquefied cortex. For the second stage capsulorhexis, anterior capsule was perforated or cut near the small capsulorhexis with a cystotome or intraocular scissors and a standard capsulorhexis was carried out approximately 5–6 mm size with an utrata. Phacoemulsification was performed in all eyes using the stop-and-chop technique with a phacoemulsification device. The rest of the surgery and in-the-bag IOL implantation were carried out using a standard technique (Video 1).

Postoperative follow-ups were done at the first day, first week and first month for all patients. Dexamethasone eye drops (Maxidex 0.1%, S.A. ALCON Couvreur N.V., Belgium), moxifloxacin eye drops (Vigamox 0.5%, Alcon Laboratories Inc. Texas, US) and nepafenac eye drops (Nevanac 0.1%, S.A. ALCON Couvreur N.V., Belgium) were used as treatment.

### Statistical analysis

The SPSS 20.0 software was used for the data analysis. Descriptive statistics were used evaluating collected data. The results were presented as mean ± standard deviation and percentage.

## Results

A total of 73 consecutive patients with intumescent cataract were evaluated in this study. There were 39 male cases and 34 female cases. Mean age was 66 years ± 8 (between 53 and 84 years). Preoperative mean BCVA was 2.34 ± 0.29 logMAR and one month median BCVA was 0.23 ± 0.27 logMAR.Preoperatively mean LT was 4.2 ± 0.5 mm and mean ACD was 2.40 ± 0.38 mm. A well centered complete continuous curvilinear capsulorhexis approximately 5–6 mm size was achieved in 72 of 73 cases (98.6%). Peripheral extension of the capsulorhexis occurred in one eye during the second stage capsulorhexis. In this case, the capsule was cut with Vannas scissors at the beginning of the capsulotomy and the capsulorhexis was completed clockwise. The rest of surgery was continued with a standard procedure and in-the-bag IOL implantation was done. The capsule was torn in two cases (2.7%) during the liquefied cortex aspiration with 25 G cannula after the first stage capsulorhexis. However, the tear did not extend to the periphery in these cases and the second stage capsulorhexis was successfully created.

## Discussion

A successful and adequate size capsulorhexis is the key step for the safe phacoemulsification and in-the-bag IOL implantation in the intumescent cataracts. In this study, we modified the two-stage capsulorhexis technique for performing a safe surgery in the intumescent cataracts. In the first stage, we created a small size capsulorhexis because the larger capsulorhexis has a greater risk of extending capsular tear to the periphery. After small size capsulorhexis, liquefied cortex was aspirated with a 25 G cannula to equalize anterior chamber pressure and intracapsular pressure. A normal size capsulorhexis was safely performed in the second stage.

It is known that cataract surgery with the one-stage continuous curvilinear capsulorhexis has higher complication rates due to the capsular tear. Therefore, the researchers tried to find new techniques to reduce the complication rates in the intumescent cataracts [4–13]. The two-stage capsulorhexis technique is one of the techniques to decrease the risk of capsular tear [6, 11–13].

To our knowledge, there are several studies about the two-stage capsulorhexis in the literature. Figueiredo et al. [6] reported Brazilian technique for prevention of the Argentinian flag sign in the white cataracts. They created a small size capsulorhexis approximately 3 mm diameter in the first stage and aspirated liquefied cortex with a bimanual irrigation/aspiration (I/A) cannulas. We believe that greater capsulorhexis size includes more risk of the capsular tear. In our study, the first stage capsulorhexis size is smaller than Figueiredo et al. study. In this study, we prefer a 25 G cannula to aspirate intracapsular liquefied cortex. We could reach the peripheral liquefied cortical material easily with this technique. It was not able to aspirate peripheral cortical material adequately using a bimanual I/A. On the other hand, Figueiredo et al. used a needle for perforating of the anterior capsule. However, we perforated the anterior capsule with an utrata and created the first stage capsulorhexis as small as possible, approximately 1.5-2 mm diameter. Utrata was not withdrawn from the anterior chamber during perforating the anterior capsule and creating capsulorhexis if the blurring of the anterior chamber did not interfere with performing the first stage capsulorhexis. This allowed us more stable anterior chamber pressure.

Vavasada et al. [11] reported two-stage capsulorhexis for intumescent cataracts. They created 3.8 ± 0.45 mm (range 3.5 to 5.1 mm) mean size of capsulorhexis in the first stage and then they performed phacoemulsification from a small size capsulorhexis. They enlarged the small capsulorhexis size before the IOL implantation in 24 eyes (%48). We believe that the small size of the capsulorhexis makes it difficult to clean the nuclear material and may increase the risk of the capsular tear during the phacoemulsification.

Kara-Junior et al. [12] reported the two-stage capsulorhexis for the intumescent cataracts. They divided the cases in two groups as one-stage and two-stage continuous curvilinear capsulorhexis techniques. The number of cases in the one-stage and two-stage groups were 13 and 11 respectively. They aspirated the liquefied cortex with the bimanual I/A after the first stage capsulorhexis in the two-stage group. However they did not state the first stage capsulorhexis size in the study. They also observed that the anterior capsular tear was 53.84% in the one-stage group and 27.27% in the two-stage group. In this small size study, there was a lower capsular tear rate in the two-stage group.

Gimbel et al. [13] reported the two-stage capsulorhexis technique. They use a 26 or 30 G needle before the first stage capsulorhexis for aspirating the liquefied milky cortex from the anterior capsulotomy area if the anterior chamber was blurred. There are also some studies that the researchers used a needle or a cannula for aspirating the liquefied milky cortex before the capsulorhexis [4, 13]. We believe that aspirating the liquefied cortex from a small capsulorhexis size is safer than only from a capsulotomy.

The modified two-stage capsulorhexis is presented for a safe cataract surgery in our study. The first stage capsulorhexis was created about 1,5 − 2 mm size in this study. To our knowledge, this is the smallest capsulorhexis size in the two-stage capsulorhexis technique. Its advantage is lower risk of capsular tear when creating the first stage capsulorhexis. The risk of capsular tear rises with increased capsulorhexis size. So, we decreased the risk of capsular tear with a small capsulorhexis size in the first stage. The liquefied milky cortex can be aspirated easily with the bimanual I/A. However, liquefied cortex that is not milky character can be aspirated mainly in the central area from a 1.5-2 mm size capsulorhexis. Thus, the peripheral intracapsular pressure does not decrease that we want. If the peripheral liquefied cortex is wanted to aspirate adequately with the bimanual I/A for a safe second stage capsulorhexis, the capsulorhexis size should be created about 3–4 mm. In this case, the capsular tear risk may increase in the first stage capsulorhexis. Therefore, we created a small capsulorhexis size about 1.5-2 mm in the first stage and we aspirated the liquefied cortex with a 25 G cannula from the peripheral intracapsular area. To our knowledge, this is the first technique for aspirating peripheral intracapsular liquefied cortex from a small capsulorhexis size with a cannula. This allowed us to aspirate not only the central liquefied cortex but also the peripheral liquefied cortex that resulted in a flatten anterior capsule. The second stage capsulorhexis is safely created with this technique according to our experiences. Consequently, we created a small capsulorhexis size in the first stage to avoid capsular tear during aspirating the intracapsular liquefied cortex totally with a cannula and this allowed us to create adequate capsulorhexis size in the second stage for a safe phacoemulsification.

In conclusion, this technique facilitates the creation of a safe capsulorhexis compared to the one-stage capsulorhexis. We also believe that this technique has aforementioned advantages over the other two-stage capsulorhexis techniques. Surgeons may consider this technique to perform a safe phacoemulsification in the intumescent cataracts.

## Electronic supplementary material

Below is the link to the electronic supplementary material.

Supplementary Material 1

Supplementary Material 2

## Data Availability

All data generated or analyzed during this study are avaliable from the corresponding author on reasonable request.
