# Supplementary material for: A safe and successful capsulorhexis technique for the intumescent cataracts; modified two-stage continuous curvilinear capsulorhexis
Source: BMC Ophthalmol. 2023 Apr 4;23:138. doi: 10.1186/s12886-023-02895-4 (PMC10074661; doi:10.1186/s12886-023-02895-4)
Supplement: Supplementary file 2 — Supplementary Material 2 [file 12886_2023_2895_MOESM2_ESM.docx]

Video 1 : The first stage capsulorhexis is performed as small as possible. The first stage capsulorhexis, aspiration of liquefied cortex with a 25 G cannula and creation of the second stage capsulorhexis are well seen in the video.
